# Genome-Wide Development and Validation of Cost-Effective KASP Marker Assays for Genetic Dissection of Heat Stress Tolerance in Maize

**DOI:** 10.3390/ijms21197386

**Published:** 2020-10-06

**Authors:** Ashok Babadev Jagtap, Yogesh Vikal, Gurmukh Singh Johal

**Affiliations:** 1 School of Agricultural Biotechnology, Punjab Agricultural University, Ludhiana 141004, India; ashok01biotech@gmail.com; 2 Department of Botany and Pathology, Purdue University, West Lafayette, IN 47907, USA; gjohal@purdue.edu

**Keywords:** heat stress, KASP, maize, RNA-seq, SNP, variant calling

## Abstract

Maize is the third most important cereal crop worldwide. However, its production is vulnerable to heat stress, which is expected to become more and more severe in coming years. Germplasm resilient to heat stress has been identified, but its underlying genetic basis remains poorly understood. Genomic mapping technologies can fill the void, provided robust markers are available to tease apart the genotype-phenotype relationship. In the present investigation, we used data from an RNA-seq experiment to identify single nucleotide polymorphisms (SNPs) between two contrasting lines, LM11 and CML25, sensitive and tolerant to heat stress, respectively. The libraries for RNA-seq were made following heat stress treatment from three separate tissues/organs, comprising the top leaf, ovule, and pollen, all of which are highly vulnerable to damage by heat stress. The single nucleotide variants (SNVs) calling used STAR mapper and GATK caller pipelines in a combined approach to identify highly accurate SNPs between the two lines. A total of 554,423, 410,698, and 596,868 SNVs were discovered between LM11 and CML25 after comparing the transcript sequence reads from the leaf, pollen, and ovule libraries, respectively. Hundreds of these SNPs were then selected to develop into genome-wide Kompetitive Allele-Specific PCR (KASP) markers, which were validated to be robust with a successful SNP conversion rate of 71%. Subsequently, these KASP markers were used to effectively genotype an F_2_ mapping population derived from a cross of LM11 and CML25. Being highly cost-effective, these KASP markers provide a reliable molecular marker toolkit to not only facilitate the genetic dissection of the trait of heat stress tolerance but also to accelerate the breeding of heat-resilient maize by marker-assisted selection (MAS).

## 1. Introduction

Maize ranks third behind wheat and rice as a staple cereal crop worldwide [1]. In terms of yield, it is one of the most productive grain crops. However, its production is negatively impacted by high temperature, which is likely to become a major stress in the future because of climate change [2,3]. Exposure to temperatures above 35 °C for a prolonged period is unfavorable for the growth and vigor of most maize germplasm in general. But the heat stress caused by high temperatures (around 40 °C and beyond) is especially damaging during flowering, reducing drastically the viability of pollen and receptivity of silks, thereby plunging grain yields [4,5]. Fortunately, some maize lines do exist that are able to withstand heat stress caused by these extreme temperatures, however, the genetic basis and mechanisms underlying these heat-resilient lines remain poorly studied. Genomic studies provide a promising tool to locate and identify genes/quantitative trait loci (QTL) responsible for the trait of heat resilience, thereby opening up opportunities to develop valuable genetic and molecular markers for marker-assisted selection (MAS) to produce climate-resilient crops. Among the different molecular markers, single nucleotide polymorphism (SNPs) are widely used in the current plant breeding programs due to their low assay cost, high genomic abundance, bi-allelic nature, locus-specificity, low mutation rate, potential for high throughput analysis, and relatively low genotyping error rates [6,7]. Hence, SNPs are the preferred markers for germplasm characterization, QTL mapping by genetic linkage or association studies, allele mining, and genomic selection studies [7,8].

The genome sequencing data obtained either experimentally from the next-generation sequencing (NGS) studies or gleaned from various databases available publicly have made it relatively simple and cheap to mine genetic variation in crop plants using various bioinformatics approaches [9]. Most of the methods detecting variation are based on sequencing data derived either from whole-genome sequencing (WGS) or whole-exome sequencing (WES) [10,11]. In the last few years, NGS approaches in the form of RNA-seq have been co-opted to provide global insights into the gene expression patterns to understand the genetic networks and metabolic pathways involved in maize responses to heat stress [12,13,14,15,16,17,18]. Apart from gene expression analysis, RNA-seq can also be used to identify genomic variants in expressed genes alongside WGS and WES [19]. In fact, RNA-seq has emerged as a cheaper and more efficient alternative to DNA sequencing platforms involving either WGS or WES [19,20]. While it remains challenging to detect genetic variants by RNA-seq because of the complexity of the transcriptome and high false positive rates [21], there are multiple advantages in carrying out SNP discovery using RNA-seq data. By providing an RNA signature or phenotype for a trait of interest, it allows genes and SNPs to be prioritized for marker development for a thorough understanding of that trait by genetic dissection [22,23]. In addition, by providing markers specifically associated with the trait of interest, it facilitates crop genetic improvement program by MAS [10,24,25].

The whole genome or transcriptome sequencing-based genome-wide SNP (variant) calling pipelines involve three steps: (i) pre-processing of the raw reads, (ii) mapping of cleaned reads to a reference genome, and (iii) identification of sequence variants (SNPs/InDels). Firstly, raw sequence reads obtained using NGS are processed by different tools to remove low quality reads and trim the adapter sequences, and only high quality (HQ) reads with the quality scores of Phred33 or Phred64 are retained. Different NGS data processing tools are available for quality check and cleaning of raw reads [26,27]. The mapping of cleaned reads to a reference genome is the next important step in variant calling pipelines. Various mappers (aligners or assemblers) are available with different algorithms and criteria for the alignment of cleaned reads to reference genomes [9,28,29]. However, the outcome of mapping is widely influenced by the choice of mapping tools and parameters [29]. Lastly, SNP callers or variant callers are used in variant calling, and single nucleotide variants (SNVs) or small insertions/deletions (InDels) can be identified [30,31]. Among the various available mappers and callers, genome-guided, splice-aware assembly mapper STAR (Spliced Transcripts Alignment to a Reference) has the highest performance compared to other mappers [28], whereas, GATK (Genome Analysis Toolkit) [30] caller has the highest accuracy in combination with most mappers [29].

The SNP genotyping data can be obtained using any of the numerous uniplex or multiplex platforms that combine a variety of chemistries, detection methods, and reaction formats [32,33]. Some common ones are TaqMan, Kompetitive Allele-Specific PCR (KASP), and rhAmp, however, the selection of the optimal platform depends on the size of the sample, number of markers, assay platform, cost-effectiveness, and accuracy [34]. The KASP uniplex assay system has gained wide popularity not only because of the advantages of having combined PCR amplification with fluorescent detection but also because of its amenability to high throughput and automation that makes it very cost-effective [7,33]. The KASP marker system has already been used for genetic and genotyping analysis in many crop plants including pigeon pea [35], chickpea [36], maize [37], wheat [38], and rice [39,40,41].

Recently, we performed RNA-seq analysis on two contrasting maize inbreds that differ in sensitivity to heat stress [42]. The inbred extremely sensitive or susceptible to heat stress was LM11, whereas the tolerant inbred was CML25. Under heat stress, the susceptible inbred LM11 exhibited top leaf firing, tassel blast, pollen sterility, and reduced pollen shedding duration, thereby resulting in small ears, reduced kernel number, kernel weight, and yield [42]. The tolerant inbred CML25 endured high heat stress without symptoms, and with no yield penalties [42]. We identified more than 2000 genes that underwent differential regulation in response to heat treatment [42]. While a number of reports have been published on transcript profiling studies aimed at identifying maize heat stress-related genes, none seem to have taken advantage of the information generated to develop KASP assay markers that could be ideally used in breeding programs. Therefore, this study was undertaken in maize with the following objectives: (i) identification of genome-wide SNP markers from transcriptomics data, (ii) development of novel KASP assays for cost-effective SNP genotyping, and (iii) validation of KASP assay markers using parental inbred lines LM11 (HS) and CML25 (HT) and an F_2_ mapping population derived from the cross of LM11 × CML25. This study reports the compilation of informative SNP data sets and the development and validation of KASP assays.

## 2. Results

### 2.1. Mapping of High-Quality (HQ) Reads to Reference Genome

The high quality (HQ) reads from the leaf, pollen and ovule RNA-seq libraries of both LM11 and CML25 were cleaned and then mapped to the B73 reference genome using assembly tool STAR 2-pass method v2.5.2b (https://github.com/alexdobin/STAR) with default parameters. A summary of all the mapped reads is given in Table 1. For LM11, the number of total cleaned reads for the leaf, pollen and ovule libraries were 27,014,405, 18,415,967, and 21,603,284, respectively. Out of these, 24,077,296 (89.13%) of the leaf, 14,657,912 (79.58%) of the pollen, and 10,875,298 (50.34%) of the ovule reads were uniquely mapped to individual loci in the B73 reference genome. In contrast, 1,291,087 (4.78%), 1,410,947 (7.66%), and 5,902,841 (27.32%) reads from the leaf, pollen, and ovule, respectively were mapped to multiple loci in the reference genome. There were a number of reads that were too short to be mapped against the B73 genome, and their proportion was 5.31%, 10.40%, and 5.47% in the leaf, pollen, and ovule libraries, respectively. In addition, some other reads that did not find counterparts in the reference genome were 0.28%, 0.26%, and 3.19% from the leaf, pollen, and ovule, respectively (Table 1).

In CML25 inbred, a total of 24,873,578, 21,310,663, and 26,208,530 cleaned reads were counted in the leaf, pollen, and ovule libraries, respectively. Out of these, 21,152,840 (85.04%), 17,387,192 (81.59%), and 21,286,482 (81.22%) reads from the leaf, pollen, and ovule, respectively were mapped to unique sites or loci in the B73 reference genome. The reads that mapped to multiple loci were 1,308,873 (5.26%), 1,438,295 (6.75%), and 2,235,321 (8.53%) from the leaf, pollen, and ovule, respectively. Among the unmapped reads from the leaf, pollen and ovule libraries, respectively, 8.86%, 10.60%, and 8.74% were too short to be matched with the reference genome. The reads that did not map for other reasons were 0.30%, 0.42%, and 0.35%, respectively, in the leaf, pollen, and ovule samples (Table 1).

The average length of the mapped forward and reverse (FR) paired-end reads was approximately 292 bp in the three libraries from both LM11 and CML25 (Table 1, Figure S1). The percentage of uniquely mapped reads in LM11 ranged from 50.34 to 89.13, whereas it ranged from 81.22 to 85.04 in CML25. The lowest number of uniquely mapped reads was from the LM11 ovule sample (50.34%), and the highest number of uniquely mapped reads (89.13%) was from the LM11 leaf sample (Table 1).

### 2.2. Variant Calling Using Genome Analysis Toolkit (GATK) SNP Caller

**Figure 1:**
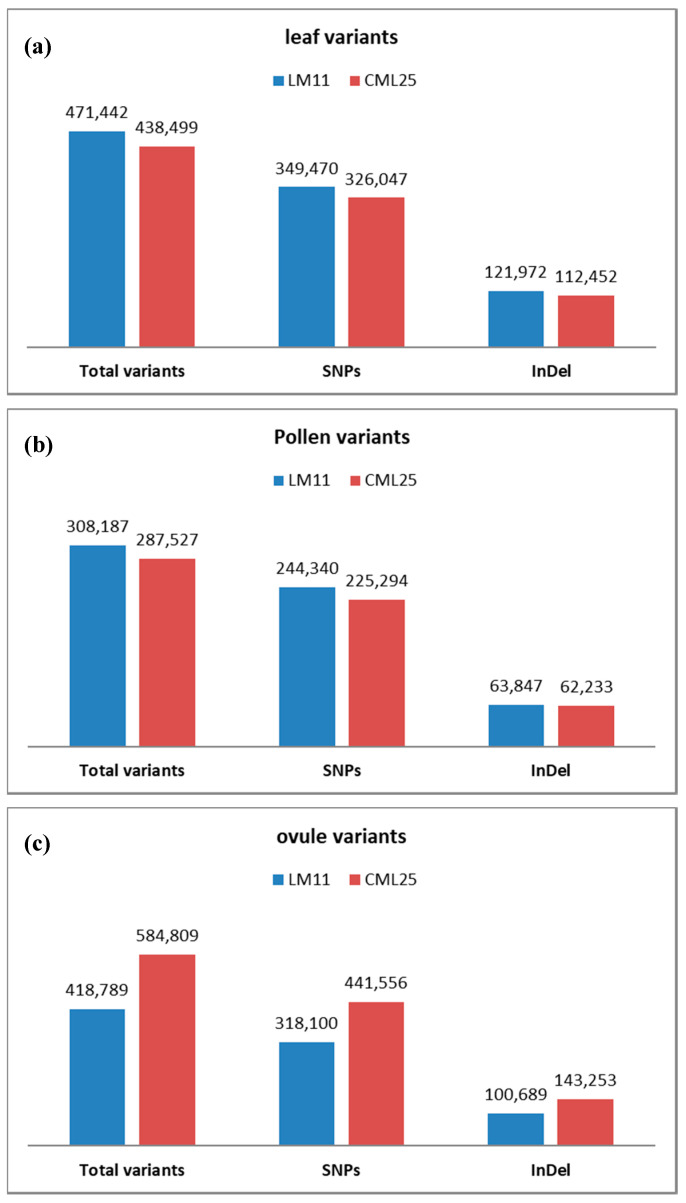
Variants identified in LM11 and CML25 with B73 reference mapping using Genome Analysis Toolkit (GATK) platform. (**a**) leaf variants, (**b**) pollen variants, (**c**) ovule variants.

On comparing the reads of the inbred LM11 with the B73 reference genome, the numbers of variants identified from the leaf, pollen and ovule libraries were found to be 471,442, 308,187 and 418,789, respectively. Out of the 471,442 variants identified from the leaf library, 349,470 were SNPs and 121,972 were InDels (Figure 1a). In the sample derived from the pollen, 244,340 of the variants were SNPs and 63,847 were InDels (Figure 1b). Likewise, out of the 418,789 variants identified from the ovule, 318,100 were SNPs and 100,689 were InDels (Figure 1c). The number of SNPs varied among the chromosomes, and the average number (variant rate) was 4461, 6819, and 5016 in the leaf, pollen, and ovule samples, respectively (Table 2).

For the other inbred (CML25), the comparison of the total reads with the reference genome identified 438,499 variants from the leaf sample; 287,527 variants from the pollen sample; and 584,809 variants from the ovule sample. Of the total variants in the leaf sample, 326,047 were SNPs and 112,452 were InDels (Figure 1a). In the pollen sample, these variants were divided into 225,294 SNPs and 62,233 InDels (Figure 1b), and in the ovule 441,556 and 143,253 variants were SNPs and InDels, respectively (Figure 1c). Again, the number of SNPs varied among the chromosomes, with the average variant rate being 4795, 7313, and 3594 in the leaf, pollen, and ovule, respectively (Table 3).

**Figure 2:**
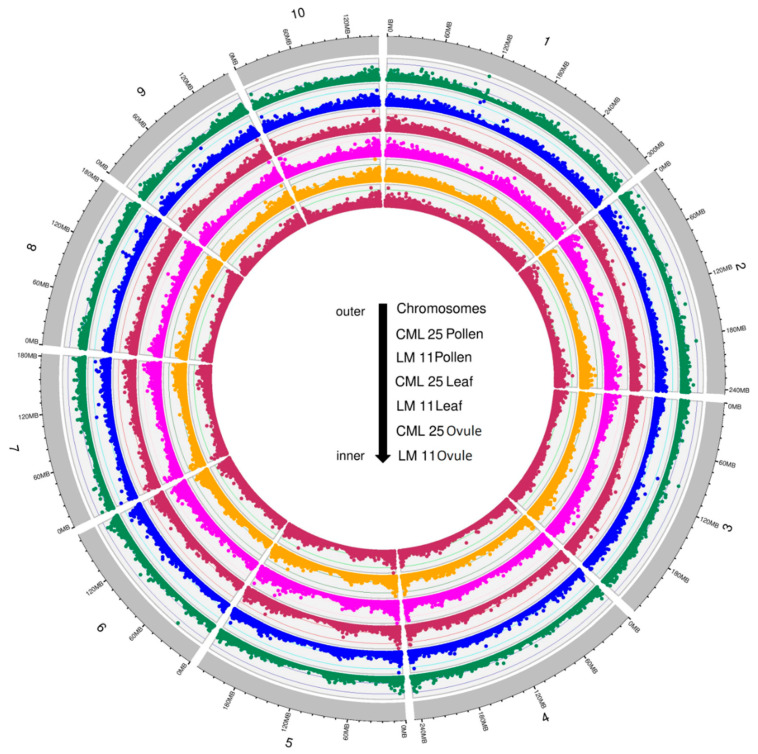
Density distributions of variants in pollen, leaf, and ovule from the CML25 and LM11 in all the 10 chromosomes of maize.

Overall, the highest number of variants was observed in the CML25 ovule library and the lowest number in the CML25 pollen library. To look into the distribution of variants across different chromosomes, the variants (SNPs and InDels) density in the leaf, pollen and ovule samples of LM11 and CML25 were plotted for each chromosome in the non-overlapping window of 1000/kb by using the R package circlize v0.4.10 (https://cran.r-project.org/web/packages/circlize/index.html) (Figure 2). Uneven distribution of variants across the 10 maize chromosomes was observed. The highest density of variants was observed in chromosome 1, whereas the lowest variant density was found in chromosome 10 in all the libraries of both LM11 and CML25 (Figure 2, Table 2 and Table 3).

**Figure 3:**
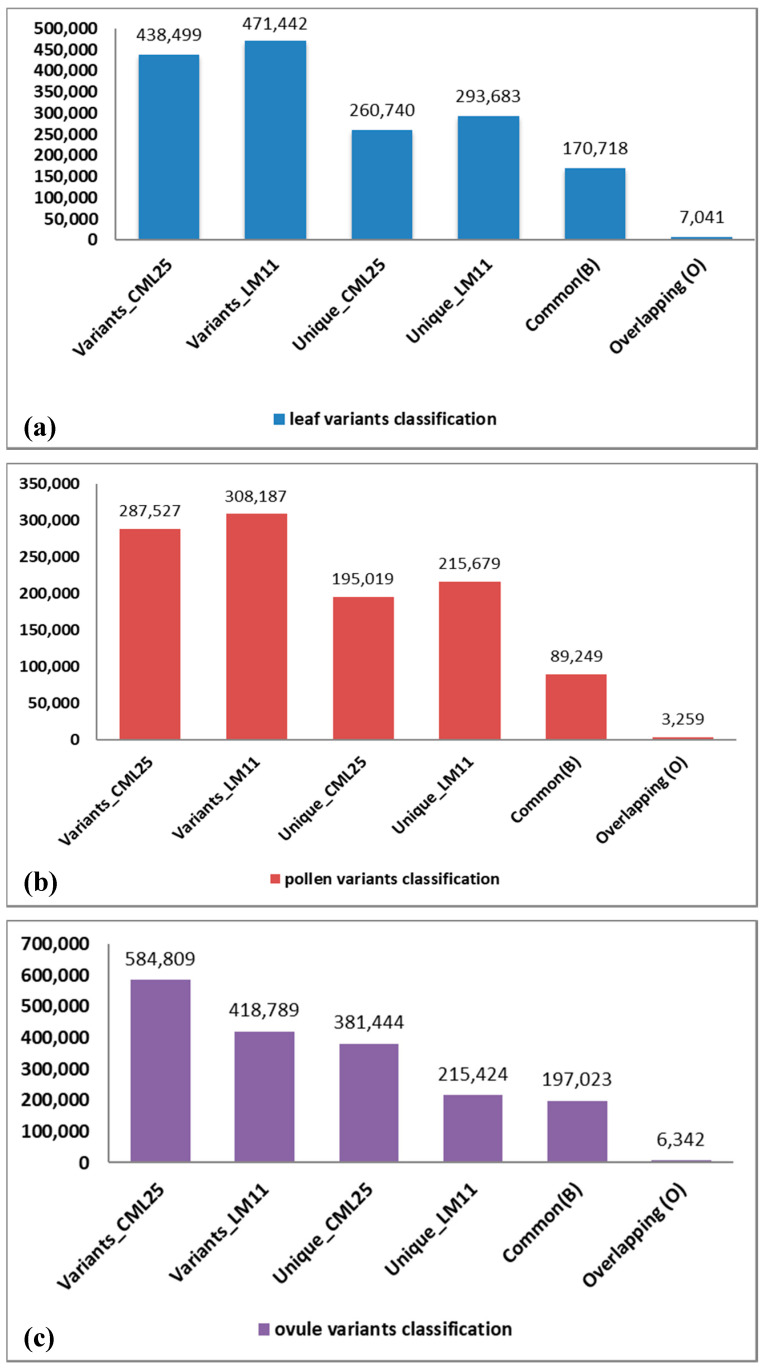
Classification of variants into different categories in CML25 and LM11. (**a**) leaf variants, (**b**) pollen variants, (**c**) ovule variants.

Although the initial mapping of the reads and variant calling was done against the reference genome of B73, our ultimate goal was to identify SNVs that are unique or specific to LM11 and CML25. Therefore, the VCFtools (https://sourceforge.net/projects/vcftools/files/) analyses were performed to compare the output Variant Call Format (VCF) file of LM11 and CML25. Based on these comparisons, the variants were categorized into three different types-unique, monomorphic, and overlapping. The unique variants are those that are specific to either LM11 or CML25. The monomorphic or common variants have the same SNP position in LM11 and CML25 but polymorphic with the B73 reference genome. The overlapping variants are the non-matching overlapping sites in LM11 and CML25 with reference to the B73 genome. Based on these criteria, the numbers of variants identified as unique to LM11 were 293,683, 215,679, and 215,424 in the leaf, pollen, and ovule libraries, respectively. Likewise, 260,740, 195,019, and 381,444 variants were unique or specific to CML25 in the leaf, pollen, and ovule, respectively. The total number of variants that were identified as monomorphic between LM11 and CML25 were 170,718, 89,249, and 197,023, respectively, in the leaf, pollen, and ovule samples. In the third category of overlapping variants between LM11 and CML25, there were 7041, 3259, and 6342 variants in the leaf, pollen, and ovule libraries, respectively (Figure 3).

### 2.3. Development and Validation of Kompetitive Allele Specific PCR (KASP) Markers

**Figure 4:**
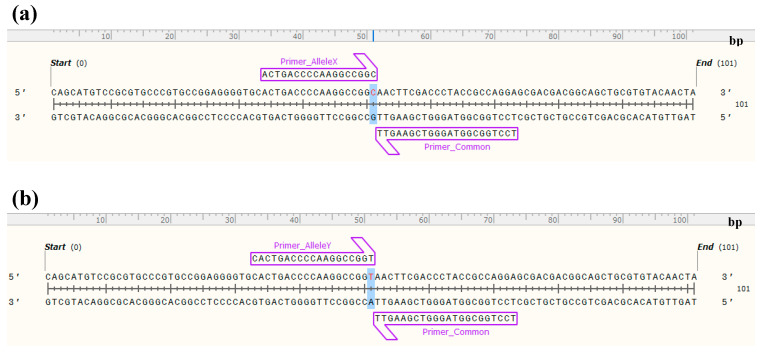
Allele specific forward and common reverse primers designed for Kompetitive Allele Specific PCR (KASP) assay marker MKAM_04_031. (**a**) allele X (LM11), (**b**) allele Y (CML25).

For the development of KASP assay markers, the polymorphic SNPs identified between the leaf libraries of LM11 and CML25 were used. These variants were filtered further to search for suitability of SNPs for KASP assay development. After filtering, out of 293,683 unique variants in the LM11 leaf, only 149,435 SNPs were retained. Likewise, out of 260,740 unique variants in CML25 leaf, only 134,491 were retained. These filtered SNPs were further subjected to more stringent selection by culling SNPs that were within 20 bp of each other. Finally, a total number of 129,804, and 117,550 SNPs were retained for LM11 and CML25, respectively. Next, a total of 100 genome-wide SNPs (10 SNPs per chromosome) were chosen to cover the entire genome for KASP assay development. The list of the KASP markers along with the flanking sequences and chromosomal locations are given in supporting data S1. The primer sequences are presented in supporting data S2, and schematic representation of KASP assay primers are presented in Figure 4.

**Figure 5:**
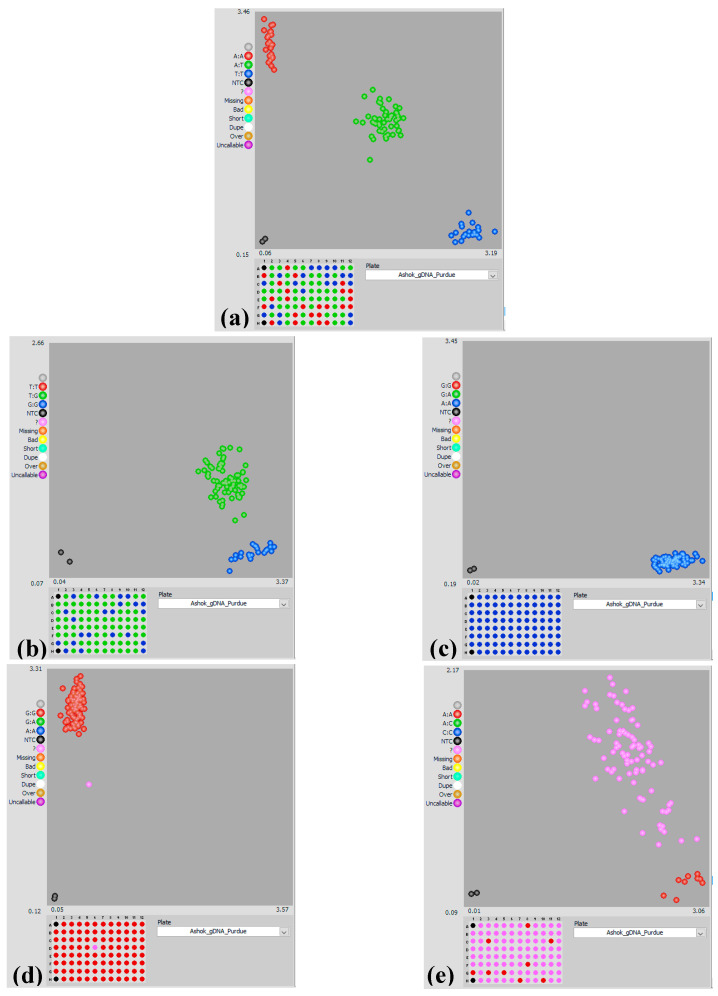
Snapshots showing single nucleotide polymorphism (SNP) genotyping in parental inbred line LM11, CML25, and their F_2_ mapping populations with KASP marker assays. The snapshots show (**a**) polymorphic pattern, occurrence of allele specific to LM11 (blue spots) and CML25 (red spots), and heterozygosity for the corresponding alleles (green spots) in F_2_ mapping population. (**b**) occurrence of only one allele (blue spots), and heterozygosity for corresponding allele (green spots). (**c**) monomorphic pattern, occurrence of allele specific to LM11 (blue spots). (**d**) monomorphic pattern, occurrence of allele specific to CML25 (red spots), and missing data (pink spot). (**e**) the majority of missing data (pink spots), and one allele (red spots), occurrence of failed amplification signals i.e., invalid markers, in all snapshots black spots represent no template controls (NTCs).

These KASP assay markers have been designated as maize KASPar assay markers, MKAMs in short. All 100 MKAMs were validated on the parental lines LM11 and CML25, and subsequently genotyped on ninety F_2_ mapping population derived from the cross LM11 × CML25, indicating that this SNP-based KASP assay markers could be used effectively in maize heat resilience breeding program. 71 (71%) of these MKAMs were found to be polymorphic, while 21 (21%) were monomorphic and/or heterozygous type and were deemed unusable as markers. The remaining 8 (8%) failed to generate a useful amplification signal and thus were unusable (Figure 5).

### 2.4. Impact Analysis and Functional Classification of SNPs

**Figure 6:**
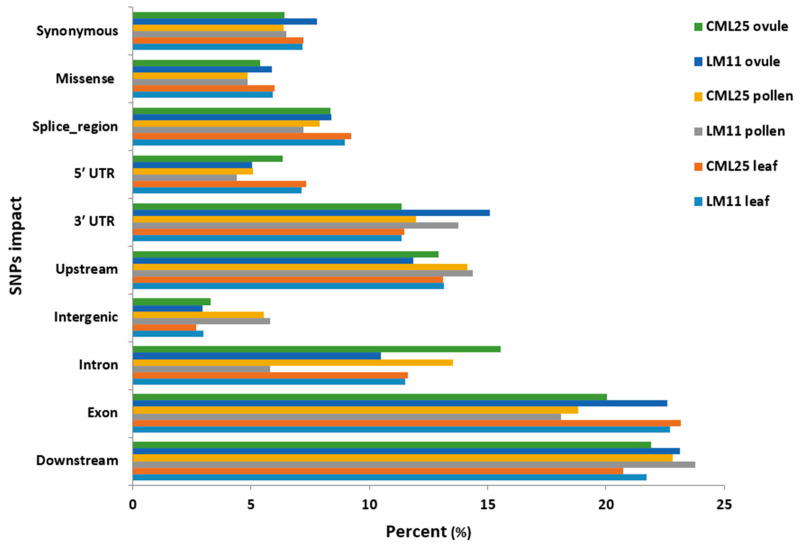
SNPs impact on the functionality of genes predicted in leaf, pollen and ovule of CML25 and LM11 using the SnpEff tool.

In terms of the types of SNP shifts, transition (Ts) and transversion (Tv) have a larger effect on the regulation of gene expression. A transition is a point mutation, which converts a purine nucleotide into another purine (A↔G) or a pyrimidine nucleotide into another pyrimidine (C↔T). Transversion refers to a purine (A or G) being substituted for a pyrimidine (C or T), or vice versa. The Ts/Tv mutation ratios of SNPs produced from GATK were 1.62, 1.83, and 1.64, respectively, in leaf, pollen, and ovule of LM11. Similarly, CML25 leaf, pollen, and ovule showed 1.64, 1.83, and 1.66 Ts/Tv mutation ratios, respectively. In our previous study, it was observed that numbers of differentially expressed genes (DEGs) identified in leaf were higher in number than pollen and ovule; and indicates leaf is most responsive to heat stress [42]. Therefore, impact analysis of identified SNPs in LM11 and CML25 leaf was performed using SnpEff tool (https://pcingola.github.io/SnpEff/). Based on the SnpEff results, SNPs of LM11 and CML25 leaf showed the highest impact compared to pollen and ovule SNPs, and it included exon followed by downstream impacts (Figure 6). Further, SNP impacts on the functionality of the genes were categorized into four types: modifier, low, moderate, and high. In LM11 leaf, SNPs with modifier (68.28%), low (12.89%), moderate (8.44%), and high (10.40%) impacts were identified. Similarly, in CML25 leaf, SNPs with modifier (67.44%), low (13.12%), moderate (8.66%), and high (10.78%) impacts were detected. The majority of SNPs in the LM11 leaf sample were identified as exonic variants (22.69%), and downstream variants (21.72%), followed by upstream (13.14%), intron (11.54%), 3ʹ UTR (11.36%), splice region (8.96%), synonymous (7.18%), 5ʹ UTR (7.12%), and missense (5.93%) variants (Figure 6). In the CML25 leaf sample, SNPs were detected as exonic variants (23.18%), and downstream variants (20.72%), followed by upstream (13.14%), intron (11.63%), 3ʹ UTR (11.47%), splice region (9.25%), synonymous (7.18%), and 5ʹ UTR (7.32%) variants (Figure 6).

### 2.5. Variants Detected in the Heat Stress Response (HSR) Genes

**Figure 7:**
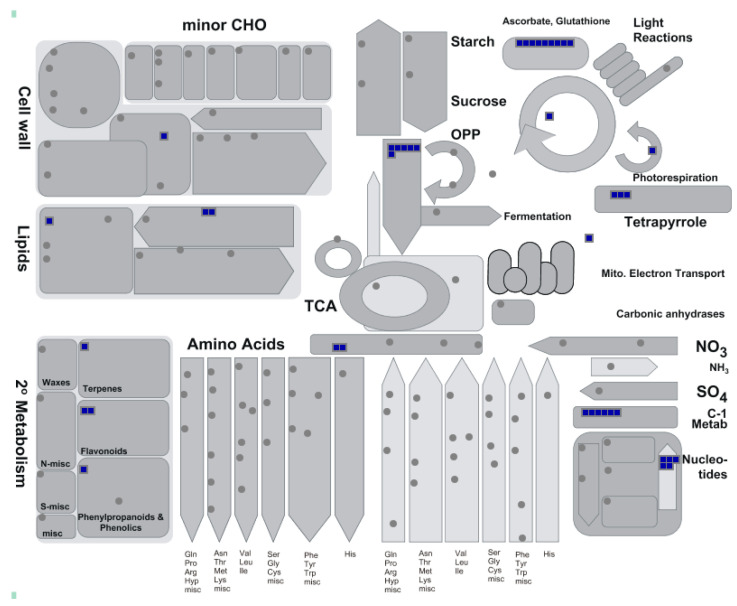
MapMan display for heat stress response (HSR) genes in LM11 and CML25. HSR genes involved in different biological functions are represented in blue color.

In this study, we observed point mutations (Ts and Tv) in the significant heat stress response (HSR) genes based on comparisons of SNP variants between LM11 and CML25. These HSR genes included transcription factors (MYB, AP2/EREBP and NAC), brassinosteroids (BRs), heat shock proteins (HSPs) *viz.* HSP70 and DNAJ, genes related to photosynthesis (Rubisco), antioxidation (APX and Glutathione S-transferase), and kinases (Table 4). Metabolic pathways analysis using MapMan tools (https://mapman.gabipd.org/) revealed that HSR genes were involved in oxidation-reduction process, response to reactive oxygen species (ROS), photosynthesis (photosystem I and chloroplast thylakoid membrane), cytochrome-c peroxidase activity, photorespiration, secondary metabolism (terpenes and flavonoids), amino acid metabolism, nucleotides metabolism, and C-1 metabolism (Figure 7).

## 3. Discussion

The discovery of single nucleotide variants (SNPs or InDels) using a RNA-seq dataset is often challenging for at least two reasons. The first reason, is that it could present a poor quality of the sequencing results, including poor read length, sequencing depth, and sequencing platforms. Second, it could also be the poor selection or operation of downstream analyses to sort SNPs [43]. We have successfully built a pipeline from using multiple bioinformatics tools for RNA-seq-based variants discovery with a high rate of accuracy. The decent sequencing quality combined with efficient bioinformatics tools allowed us to develop an excellent set of genome-wide and cost-effective KASP assay markers for high-throughput SNP genotyping of the maize germplasm for heat stress resilience. The choice of SNP mapper and caller are crucial in downstream analyses of RNA-seq data for SNP discovery. Our study demonstrated that the combination of STAR mapper and GATK variant caller are the best performers for RNA-seq based single nucleotide variants discovery.

Our findings are in line with a few recent reports showing the appropriate use of STAR mapper and GATK variant caller for SNP discovery from RNA-seq data [9,28,29,43]. For instance, Zhao et al. [9] described a high throughput SNP discovery strategy for RNA-seq data from peach and mandarin. Their study constituted a comprehensive comparison of two paired-end read lengths (125 bp and 150 bp), five assemblers (Trinity, IDBA, oases, SOAPdenovo, Trans-abyss), and two SNP callers (GATK and GBS). They observed that the rate of false positive SNPs was significantly lower when the paired-end read length was 150 bp compared with 125 bp. Trinity was found to be superior to the other four assemblers that they used, and GATK was significantly superior to GBS due to a low rate of missing authentic SNPs. This combination of the assembler Trinity, SNP caller GATK, and the paired-end read length of 150 bp had the best performance in SNP discovery with 100% accuracy both in peach and mandarin.

Similarly, Tanaka et al. [43] used GATK SNP caller for the development of genome-wide SNP markers in barley via reference-based RNA-seq analysis. They used 150 samples from 108 strains (accessions) for this study. A total of 181,567 SNPs and 45,135 InDels located in the 28,939 transcribed regions and distributed throughout the barley Morex reference genome were detected. They further evaluated the quality of this polymorphism detection approach by analyzing 387 RNA-seq-derived SNPs using amplicon sequencing. More than 85% of the RNA-seq SNPs were validated using the highly redundant reads from the amplicon sequencing, and this demonstrated the accuracy of GATK caller. Likewise, using GATK caller, we discovered a total of 554,423, 410,698, and 596,868 SNVs between LM11 and CML25 after comparing the RNA-seq transcript reads of leaf, pollen, and ovule, respectively. This RNA-seq-based SNPs discovery approach led us to develop 100 KASP markers (MKAMs), and these were validated to work with a 92% success rate using the parental inbreds LM11 and CML25, and their F_2_ population, indicating the high accuracy of GATK caller.

Variant calling pipeline with a high sensitivity or high specificity is thus essential to accelerate the power of analyses by identifying true positive variants [29,44]. An evaluation of seven different RNA-seq alignment tools, BWA, CLC, HISAT2, Kallisto, RSEM, Salmon, and STAR on RNA-seq data from the model plant *Arabidopsis thaliana* showed that mapper BWA had the lowest performance and STAR had the highest [28]. In another report, GATK in combination with CLC-mapper, Novoalign, and BWA-MEM yielded the best and most consistent results across all a number of datasets of *A. thaliana* that were evaluated [29]. In addition, the rate of authentic variant identification from RNA-seq data depends on the detection of true splice junctions (SJ). The splice junction detection is significantly improved by paired-end and longer read length (>100 bp) [45] and the splice-aware assembly tool STAR [20]. Our results are in line with previous studies, and we found that the combination of assembler STAR and SNP caller GATK with paired-end read length of 150 bp showed the best performance.

For SNP calling and discovery to be useful for practical plant breeding applications, low-cost, breeder-friendly predictive markers are needed to develop from these polymorphic sites for marker-assisted selection in small-scale breeding programs. Different platforms are available to detect and genotype SNPs, and their selection relies on numerous factors such as the precision, reproducibility, high-throughput, and multiplexing, as well as the time and cost-effectiveness of the protocol used [7]. In this investigation, we demonstrate the convenience of using RNA-seq data to develop the SNP-based KASP assay markers, and how they were subsequently used in a timely and cost-effective manner to genetically dissect the trait of heat stress tolerance in maize. Several reports have been published on the development of KASP assays in other plants as well. The SNP based KASP analysis has been successfully used in pigeon pea [35], chickpea [36], maize [37], wheat [38], tomato [46], rice [40,41,47], and radish [7] for diversity analysis, genetic purity test, quality control, cultivar discrimination, and economic trait advancement to enhance molecular breeding. A comparison involving genotyping of 100 chickpea lines with 500 SNPs using GoldenGate and KASP assays showed that the KASP assays were superior to GoldenGate assays in terms of both cost and time [36]. In summary, all these studies address the utility of KASP assays for SNP genotyping on a large scale with low error rates and cost-effectiveness. In the present study, 100 genome-wide SNP sites were selected for KASP assay designing from SNPs captured using RNA-seq data. All selected 100 SNP sites (100%) were converted to KASP assay markers. Out of 100 KASP markers, a total of 71% were polymorphic and were deemed usable as markers. A total of 21% KASPs were generated only one or heterozygous type genome call and were deemed unusable as markers, and 8% failed to generate a useful amplification signal (unusable type). The failure of the remaining SNP markers (29%) could be due to the presence of paralogous sequences, incorrect primer designing near the SNP, identification of false or unreal SNPs initially, and/or the need to optimize PCR conditions. This conversion rate (71%) was higher than that of the other KASP studies on wheat (67%), rice (49.9%) but lower than in chickpea (80.6%).

Recently, Yang et al. [47] developed KASP markers using SNP information from DNA sequencing data available publicly on international rice genome databases. A total of 565 out of 596 SNP sites were successfully converted to KASP markers with a 94.8% success rate of assay designing. This rate of conversion from selected SNPs to functional KASP assays could probably be increased with optimization of primer designing and amplification conditions. However, we have made no attempt to optimize the failed assays. This emphasizes the need for stringent selection criteria and the validation of in silico identified SNPs via allele re-sequencing. The number of polymorphic KASP markers identified in our study resulted in a much lower genotyping error rate than that obtained with markers such as SSRs.

The SNP conversion rate is composed of two components—the design success rate and the work success rate. The ratio of the SNP sites that can be used to design primers to the total number of SNP sites is called the design success rate. The work success rate refers to the number of SNP sites that can generate genotype calls via primers to the number of SNP sites with successfully designed primers [8]. Two studies in rice, which used DNA sequencing data, and reported the KASP assay design success rate of 49.9% [41] and 94.8% [47], whereas their work success rate was 96.6% and 93.6%, respectively. In our study, we developed KASP markers with an assay design success rate of 100% and a work success rate of 92%. In addition, we used RNA-seq data which, compared to the DNA sequencing data, exhibits higher sensitivity and specificity in SNP calls. The KASP assay design and work success rate in our study are thus in accordance with previous reports by Cheon et al. [41] and Yang et al. [47].

The impacts of SNPs on the function of genes they are in can be considered high, low, or moderate (also termed modifiers) [48]. High impact SNPs usually cause a gain or loss of a stop codon, thereby resulting in a major change in the function of a gene. SNPs are low impact when they have a minimal or no impact on the function of a gene, and such SNPs often cause synonymous changes in which a nucleotide change does not translate into an amino acid change. The moderate impact or modifier SNPs result in missense mutations and thus can dampen or modify the function of a gene. Modifier SNPs are often in the coding region but can also be present in the 3ʹ or 5ʹ UTR. In the present study, the SNPs with the modifier effects were identified predominantly as the exonic, downstream, and upstream variants in both LM11 and CML25. Similarly, Kim et al. [7] studied SNPs impact on the functionality of genes using SnpEff. In their study, most SNPs with the modifier effect were observed as downstream gene variants (29.7%), upstream gene variants (27.0%), and synonymous variants (18.3%).

In response to heat stress, plants accumulate different metabolites such as antioxidants, osmoprotectants, and HSPs that trigger multiple pathways involved in signal transduction, the scavenging of ROS, and maintaining cell membrane stability to improve damage and sustain cell homeostasis [49]. In the recent past, many studies have been identified and analyzed HSR genes in maize [12,13,14,15,16,17,18]. For example, in our previous study, we were observed differential expression of HSR genes related to transcription factors *viz*. AP2, MYB, WRKY, PsbP, bZIP and NAM, HSPs (HSP20, HSP70, and HSP101/ClpB), photosynthesis (PsaD and PsaN), antioxidation (APX and CAT), BRs, and polyamines (Spd and Spm) in LM11 and CML25 under heat stress treatment [42]. In this study, we identified SNP variants in reported HSR genes between LM11 and CML25 which indicates that these SNP variations may be responsible for altering metabolic pathways in these two inbred lines under heat stress.

## 4. Materials and Methods

### 4.1. Plant Materials and DNA Extraction

**Figure 8:**
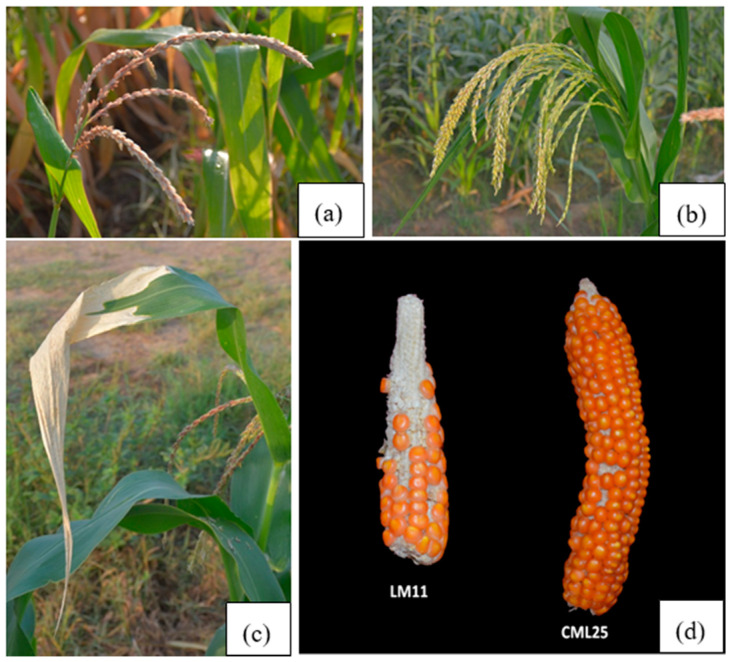
The impacts of heat stress on various phenological attributes between LM11 and CML25. (**a**) tassel blast in LM11, (**b**) no tassel blast or top leaf firing in CML25, (**c**) top leaf firing in LM11, (**d**) variations in kernel number and ear size.

Two inbred lines contrasting for heat stress tolerance used were CML25, tolerant; and LM11, sensitive. The F_2_ mapping population was derived from the cross of LM11 (HS) and CML25 (HT). Both inbreds possessed contrasting phenological attributes comprising of top leaf firing, tassel blast, pollen viability, pollen shedding duration, kernel number, kernel weight, and yield (Figure 8) [42]. These two lines and derived ninety F_2_ mapping populations were raised in a glasshouse using standard agronomic practices at the School of Agricultural Biotechnology, Punjab Agricultural University, Ludhiana, Punjab, India. Genomic DNA was extracted from 20-days old seedlings leaves of parental inbred LM11 (HS) and CML25 (HT) and derived F_2_ mapping population using the cetyl trimethylammonium bromide (CTAB) method [50]. The DNA which had RNA contaminant was purified by RNase treatment given with 4 µL of RNase (10 mg/mL) and incubated at 37℃ for 30 min. Agarose gel (0.8 g/100 mL) electrophoresis was used to compare the light intensity of DNA samples with the known concentration (10, 20, 50, and 100 ng/μL) of standard lambda DNA. DNA samples were normalized to 10 ng/μL for each sample using NanoDrop^®^ ND-1000 spectrophotometer (NanoDrop Technologies, Inc., Wilmington, DE, USA).

### 4.2. Pre-Processing of RNA-Seq Data and Variants (SNP and InDels) Discovery

**Figure 9:**
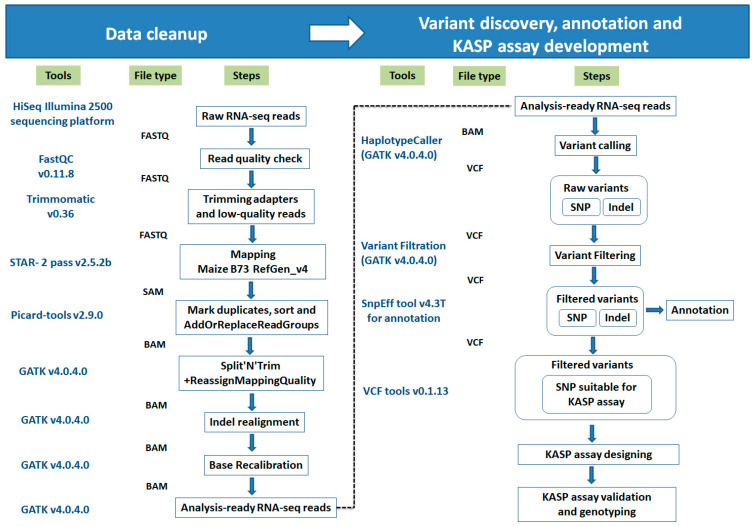
Variant calling pipeline. Schematic representation of the bioinformatics pipeline for variant calling, annotation, KASP assay development and genotyping from RNA sequencing reads.

We have schemed variant calling and KASP assay development pipelines for RNA-seq data (Figure 9). Each step of this pipeline is described hereafter.

RNA-seq of the top leaf, pollen, and ovule from LM11 (HS) and CML25 (HT) at reproductive stage under heat-stressed conditions were performed using Illumina HiSeq 2500 sequencing platform (Illumina, San Diego, CA, USA), and it was outsourced from Nucleome Informatics Pvt. Ltd., Hyderabad, Telengana, India. RNA-seq raw reads were processed with FastQC v0.11.8 [26] to check the quality. Trimmomatic v0.36 [27] were used to remove low-quality regions and adapter fragments; with options 2:30:10 and sliding window size kept as four to trim the reads having average PHRED score below 20. Reads pairs with only one surviving read and reads with a cut-off value below 100 nt were discarded. At the same time, FastQC performed on cleaned read, and Q20, Q30, and GC contents of the clean data were calculated. All downstream analyses were based on high quality (HQ) cleaned reads.

RNA-seq high quality (HQ) cleaned read data from the top leaf, pollen, and ovule of LM11 (HS), and CML25 (HT) was exploited for variant identification. The RNA-seq HQ cleaned reads exported in FASTQ format were mapped with the maize reference genome (B73 RefGen_v4, MaizeGDB database) using the splice-aware assembly tool STAR v2.5.2b 2-pass method with default parameters [51]. STAR is considered as the most accurate mapper for RNA-seq reads and can detect splice-junctions [20,52]. It performs the first mapping pass (mapping pass 1) to compares the reads with the reference genome encoded in a static genome index file. The first mapping pass produced a SAM output file that contains the mapping location of each read to the reference; but it did not take into consideration that these RNA reads are spliced (i.e., split) at multiple intermediate locations (so-called junctions). The SAM output file produced after mapping pass 1 was discarded since the mapping was not accurate. In addition to the SAM file, the first mapping pass produced a splice junction (SJ) file that contains the splice junction information. STAR used that SJ file as a guide in the rebuilding of the genome index (rebuild genome index), which produced a new genome index with splice junction information. Then, STAR starts a second mapping pass (mapping pass 2) to perform a more accurate mapping of the spliced reads in the FASTQ file which again created an accurate SAM file with read mapping information.

An analysis tool, SAMtools v1.4.1 [31] was used to convert the SAM mapping file to BAM format. The BAM files were further processed for sorting, adding read groups, and marking of duplicates reads using the Picard tools v2.13.2 package (https://broadinstitute.github.io/picard/). Subsequently, the widely used SNP caller, GATK v4.0.4.0 [30] were applied to perform prediction of variants (SNP or InDels). The SNP calling step uses the GATK toolkit i.e., splitting “N” cigar reads (splice junction reads), BaseRecalibrator (base quality score recalibration), HaplotypeCaller (variants detection) and VariantFiltration (variants filtration) [30]. The location of gaps in RNA reads was identified by the Split’N’Trim tool, and then subsequently split a spliced read into exon segments. After that, the BaseRecalibrator tool was used to reassign the base quality values of the reads in table format that could be biased by the sequencing machines. Finally, the HaplotypeCaller tool was applied for variant calling using the BAM file and the base quality table, and probable variants in the reads with respect to the reference were identified and wrote into a variant call file (VCF) format. After that, detected variants i.e., found in mapping plus SNP calling steps were filtered out to minimize false positive variant calls and considered them as priority SNPs. The priority SNPs were filtered with the set of read characteristics summarized by Adetunji et al. [10] using the GATK VariantFiltration tool. In addition, after GATK VariantFiltration, VCFtools v0.1.13 [53] were used to further filter out specific variants. The VCFtools allowed summarizing variants, converted them into different file types, validated and merged files, created intersections and subsets of variants [53]. The VCFtools option “--remove-filtered-all” was used for elimination of all sites with a filter flag other than “PASS”. Lastly, variant call file (VCF) of inbreds LM11 (HS) and CML25 (HT) were compared using VCFtools with the option “--diff-site” and polymorphic SNPs between inbreds LM11 (HS) and CML25 (HT) were identified, and were further handled for KASP marker development. The filtered SNP variants were annotated with SnpEff v4.3T [54] tool using the maize B73 RefGen_v4 reference genome to determine the effects of the SNPs on the function of genes. The distribution of SNP variants on each maize chromosome was visualized in the non-overlapping window of 1000/kb using the R package circlize v0.4.10 [55], and a circos plot was created using the R package RLumShiny v0.2.2 [56]. Biological pathway analysis of HSR genes was carried out using MapMan v3.6.0RC1 [57].

### 4.3. Variant Filtering for Designing KASP Assay Markers

Variant call format files processed by VCFtools were parsed using in-house customized Perl script to retrieve the flanking sequences 50 bp either side of each variation site and variants suitable for KASP markers were screened following a stepwise identification process [40]. The criteria for selection were that the flanking sequences (a) did not contain any InDels, (b) contained a maximum of four ambiguous bases, (c) had a base coverage of at least five at any position, (d) had no more than four consecutive repeats of any one to five nucleotide sequences, and (e) SNP in homozygous condition. Variants that passed this filtering were defined as potential KASP markers. The SNP positions of the potential KASP markers were used in the genotyping of parental inbreds i.e., LM11 and CML25, and derived F_2_ mapping population of the cross LM11 (HS) × CML25 (HT).

### 4.4. Development of KASP Assay Markers

For the development of potential KASP assay markers, genomic locations of DEGs that we identified in our previous transcriptome study of the LM11 and CML25 was used as streamline [42], and a total of 100 SNPs site spanning all 10 chromosomes of maize were selected from leaf variants. The SNP sequence, 50 bp left-flanking sequence and 50 bp right-flanking sequence of each SNP site were used to design two allele-specific forward primers carrying the standard FAM (5′ GAAGGTGACCAAGTTCATGCT 3′) and HEX (5′ GAAGGTCGGAGTCAACGGATT 3′) tails and with the targeted SNP at the 3′ end and a common reverse primer. The design and manufacture of the KASP marker primers were performed by LGC, Biosearch Technologies (Beverly, MA, USA), and ordered KASP primer oligos from LGC, Biosearch Technologies. The designed KASP assay markers were visualized using SnapGene Viewer v4.2 (GSL Biotech LLC; http://www.snapgene.com/).

### 4.5. Validation of KASP Markers and Subsequent Use in Genotyping

Genotyping reaction reagents were purchased from the LGC, Biosearch Technologies (Beverly, MA, USA). A total of 100 KASP assays were developed and validated on DNA of ninety F_2_ individuals, parental DNA of LM11 (HS) and CML25 (HT) in duplicates and two non-template controls (NTC). Assays were tested in 96-well formats with reaction set up of 10 μL [2 μL wet DNA (10 ng/μL final concentration of DNA), 5 μL of 2X KASP master mixture, 0.14 μL of assay mix and 2.86 μL nuclease-free water]. PCR cycling was performed in Flex Real-Time PCR System (Applied Biosystems, Foster City, CA, USA) using the following conditions: hot start for 15 min at 95 °C, followed by ten touchdown cycles (20 s at 95 °C; touchdown at 65 °C initially and decreasing by −1 °C per cycle for 25 s), followed by 30 additional cycles of annealing (10 s at 95 °C; 60 s at 57 °C). An extension step was unnecessary as amplicons are usually less than 100 bp long. The plate was read using a Tecan Safire plate reader (TecanGroup Ltd., Männedorf, Switzerland) at room temperature.

### 4.6. Scoring of KASP Markers Data

Data analysis was performed manually using Klustercaller v2.22.0.5 software (LGC, Biosearch Technologies, Beverly, MA, USA). Based on the fluorescence signal, the SNP allele call data were graphically illustrated for individual markers assayed using the SNPviewer software (LGC, Biosearch Technologies, Beverly, MA, USA).

## 5. Conclusions

In conclusion, the results presented in this paper reveal that large-scale SNP identification through RNA sequencing is an attractive approach, and it has facilitated the development of a set of robust and cost-effective KASP markers for maize inbreds LM11 and CML25. To our knowledge, this study is the first report on SNP based KASP assay development for heat stress-related responses in maize using RNA-seq data. These KASP markers will be helpful in conducting mapping studies and underpinning genes/QTLs for heat stress adaptive traits, and will ultimately accelerate the breeding of heat resilient maize cultivars.

## Figures and Tables

**Table 1:** Summary statistics of LM11 and CML25 reads mapped to B73 reference genome.

Parameters	LM11			CML25		
	Leaf	Pollen	Ovule	Leaf	Pollen	Ovule
Number of input reads	27,014,405	18,415,967	21,603,284	24,873,578	21,310,663	26,208,530
Average input read length (bp)	292	292	292	291	292	291
Uniquely mapped reads number	24,077,296	14,657,912	10,875,298	21,152,840	17,387,192	21,286,482
Uniquely mapped reads (%)	89.13	79.58	50.34	85.04	81.59	81.22
Average mapped length (bp)	288.67	287.76	288.43	287.61	288.06	287.17
Number of reads mapped to multiple loci	1,291,087	1,410,947	5,902,841	1,308,873	1,438,295	2,235,321
Reads mapped to multiple loci (%)	4.78	7.66	27.32	5.26	6.75	8.53
Reads unmapped: too short (%)	5.31	10.40	5.47	8.86	10.60	8.74
Reads unmapped: other (%)	0.28	0.26	3.19	0.30	0.42	0.35

**Table 2:** Chromosome-wise distribution of variants in LM11.

Chromosome	Length (bp)	LM11 Leaf		LM11 Pollen		LM11 Ovule	
		Variants	Variant Rate	Variants	VariantRate	Variants	Variant Rate
1	307,041,717	73,811	4159	46,237	6625	65,232	4694
2	244,442,276	56,785	4304	35,385	6895	49,689	4905
3	235,667,834	51,722	4556	33,505	7019	44,373	5297
4	246,994,605	44,735	5521	32,385	7613	41,052	6002
5	223,902,240	54,162	4133	35,380	6310	48,434	4608
6	174,033,170	39,028	4459	26,073	6659	35,112	4943
7	182,381,542	38,371	4753	24,981	7284	35,189	5170
8	181,122,637	43,402	4173	27,336	6610	38,038	4749
9	159,769,782	35,597	4488	24,002	6640	32,400	4917
10	150,982,314	34,529	4372	22,903	6579	29,270	5143
Total	2,106,338,117	472,142	4461 *	308,187	6819 *	418,789	5016 *

* Average variant rate.

**Table 3:** Chromosome-wise distribution of variants in CML25.

Chromosome	Length (bp)	CML25 Leaf		CML25 Pollen		CML25 Ovule	
		Variants	Variant Rate	Variants	Variant Rate	Variants	Variant Rate
1	307,041,717	69,000	4439	44,550	6878	91,423	3348
2	244,442,276	51,818	4711	32,465	7518	70,413	3464
3	235,667,834	48,352	4865	31,409	7489	64,403	3653
4	246,994,605	41,606	5923	29,443	8372	56,537	4359
5	223,902,240	51,231	4363	32,303	6922	67,852	3293
6	174,033,170	36,881	4709	24,613	7057	49,337	3517
7	182,381,542	35,354	5152	24,164	7538	46,977	3874
8	181,122,637	40,237	4492	26,764	6755	52,739	3429
9	159,769,782	32,948	4843	21,679	7361	43,906	3633
10	150,982,314	31,072	4850	20,137	7487	41,222	3656
Total	2,106,338,117	438,499	4795 *	287,527	7313 *	584,809	3594 *

* Average variant rate.

**Table 4:** Heat stress response (HSR) genes with SNP variants in LM11 and CML25.

Gene Id	Chromosome	SNP Position	LM11 Allele	CML25 Allele	Gene Descriptions
Zm00001d028325	1	30799147	G	C	brs1;brassinosteroid synthesis1
Zm00001d029149	1	60531451	A	G	Zinc finger protein CONSTANS-LIKE 13
Zm00001d029892	1	92734626	T	C	Metalloendoproteinase 1-MMP
Zm00001d033805	1	274242109	G	A	Glutamate decarboxylase 1
Zm00001d002597	2	16399614	T	G	Rho GTPase-activating protein 3
Zm00001d003643	2	51510367	A	G	L-ascorbate peroxidase S chloroplastic/mitochondrial
Zm00001d006036	2	195894333	G	C	Heat shock 70 kDa protein 9 mitochondrial
Zm00001d041701	3	133891803	G	A	Acyl carrier protein 2 chloroplastic
Zm00001d048592	4	920844	C	T	rca2; RUBISCO activase2: encodes the beta form of RUBISCO activase
Zm00001d051056	4	139237665	T	A	S-adenosylmethionine decarboxylase proenzyme
Zm00001d017729	5	205460426	C	T	Serine/threonine-protein kinase MHK
Zm00001d017992	5	211554167	T	G	Metalloendoproteinase 1
Zm00001d037273	6	119044168	T	C	Peptide methionine sulfoxide reductase msrB
Zm00001d037663	6	133420821	C	T	NADH-ubiquinone oxidoreductase 10.5 kDa subunit
Zm00001d039188	6	172189672	A	T	Putative leucine-rich repeat receptor-like protein kinase family protein
Zm00001d008546	8	12514212	C	G	Putative AP2/EREBP transcription factor superfamily protein isoform
Zm00001d010227	8	104777615	A	T	Putative NAC domain transcription factor superfamily protein isoform
Zm00001d011760	8	160944801	A	G	DNAJ heat shock N-terminal domain-containing protein
Zm00001d044785	9	2409913	A	G	putative MYB DNA binding family protein-G2-like 1
NIP2-3	9	4090209	T	G	aquaporin NOD26-like membrane integral protein ZmNIP2-3
Zm00001d045220	9	16482063	C	A	Late embryogenesis abundant protein group 2
Zm00001d046363	9	84132237	C	T	S-adenosyl-L-methionine-dependent methyltransferases superfamily protein

## Supplementary Materials

Supplementary Materials can be found at https://www.mdpi.com/1422-0067/21/19/7386/s1. Figure S1: Statistical distribution of fragment lengths. (a) leaf CML25, (b) leaf LM11, (c) pollen CML25, (d) pollen LM11, (e) ovule CML25, (f) ovule LM11, Supporting data S1: List of genome-wide KASP assay markers developed using RNA-seq data, Supporting data S2: List of KASP primer sequences (two forward and one reverse) designed for LM11 and CML25 alleles.

## Abbreviations

BRs	Brassinosteroids
DEGs	Differentially expressed genes
GATK	Genome Analysis Toolkit
GBS	Genotyping-by-sequencing
HSPs	Heat shock proteins
HS	Heat stress susceptible
HSR	Heat stress response
HT	Heat stress tolerant
KASP	Kompetitive allele specific PCR
MAS	Marker-assisted selection
MKAMs	Maize KASPar Assay Markers
NGS	Next-generation sequencing
ROS	Reactive oxygen species
SNP	Single nucleotide polymorphism
SNVs	Single nucleotide variants
STAR	Spliced Transcripts Alignment to a Reference
WES	Whole exome sequencing
WGS	Whole genome sequencing
