# Supplementary material for: Genome-Wide Development and Validation of Cost-Effective KASP Marker Assays for Genetic Dissection of Heat Stress Tolerance in Maize
Source: Int J Mol Sci. 2020 Oct 6;21(19):7386. doi: 10.3390/ijms21197386 (PMC7582619; doi:10.3390/ijms21197386)
Supplement: Supplementary file 1 [file ijms-21-07386-s001.zip › SupplementaryData/FigureS1.docx]

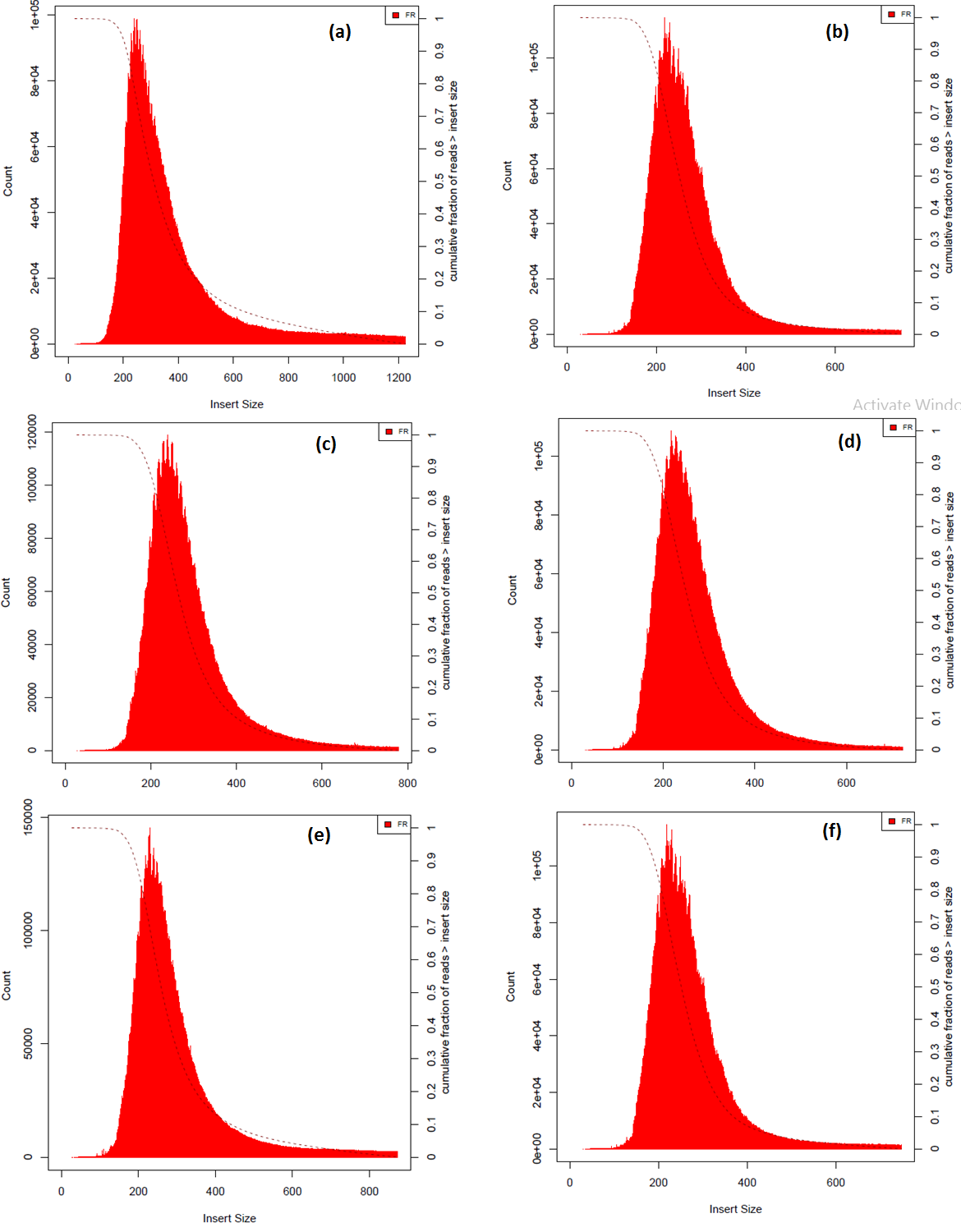


**Figure S1:** Statistical distribution of fragment lengths. (a) leaf CML25, (b) leaf LM11, (c) pollen CML25, (d) pollen LM 11, (e) ovule CML25, (f) ovule LM11.
